# A Scoping Review of Australian Aboriginal Early Relational Health Knowledge Systems

**DOI:** 10.1007/s10567-026-00570-w

**Published:** 2026-05-02

**Authors:** Shannon McNeair, Catherine Chamberlain, Naomi Priest, Tracy Evans-Whipp, Suzanne Vassallo, Kayla Mansour, Lisa Ritland, Craig A. Olsson, Juli Coffin, the Australian Early Relational Health Network

**Affiliations:** 1 School of Psychology, Deakin Institute for Lifespan Health and Development, Faculty of Health, Deakin University, Victoria, Australia; 2 Indigenous Health Equity Unit, Melbourne School of Population and Global Health, University of Melbourne, Victoria, Australia; 3 POLIS@ANU: The Centre for Social Policy Research, Australian National University, Acton, Australia; 4 Human Early Learning Program, School of Population and Public Health, University of British Columbia, Vancouver, Canada; 5 Murdoch Children’s Research Institute, Melbourne Royal Children’s Hospital, Victoria, Australia; 6 Ngangk Yira Institute for Change, Murdoch University, Murdoch, WA Australia; 7 Nulungu Research Institute, Notre Dame University, Fremantle, Australia; 8 The University of Western Australia, Crawley, Australia

**Keywords:** Early relational health, Aboriginal, Cultural practices, Indigenous, Scoping review

## Abstract

Australian Aboriginal people have evolved advanced relational knowledge systems for raising children and young people over thousands of years. Documentation of this knowledge within peer-reviewed literature is dispersed across a broad set of disciplines and sources that are mostly contextualised within Eurocentric frameworks. In this scoping review, we describe the literature on Australian Aboriginal Early Relational Knowledge Systems. Our aim is to describe how Australian Aboriginal approaches to relational wellbeing uniquely promote health and human development from pre-conception, to childhood, to adolescence and into parenting next generation offspring. We privilege Australian Indigenous authors as the holders of knowledge and limit our review to data sources led by Australian Indigenous people. A systematic search for relevant studies was conducted across four databases: MEDLINE, PsycINFO, Embase and CINAHL. This yielded 5,141 articles, of which 26 met criteria. A further 5 articles were identified through citation searching resulting in 31 included articles. We describe how Aboriginal children and young people develop relational security through engagement with multiple complex social systems within family and community, through interaction with Country, and across human and non-human elements, many features of which are missed in Western theories and Western interpretation of Aboriginal relational knowledge. Culturally valid and reliable assessment measures are urgently needed to accurately represent and monitor population relational health in Aboriginal children and young people.

**Supplementary Information:**

The online version contains supplementary material available at 10.1007/s10567-026-00570-w.

## Introduction

Early Relational Health is a term increasingly used to define the extent to which the relational context around the developing child is able to meet fundamental human needs for security and growth across the earliest years of life (National Academies of Sciences & Medicine, 2025; Olsson et al., 2026). Aboriginal and Torres Strait Islander people are the oldest living culture in the world, with ancient knowledge systems that have provided generations of extensive collectivist knowledge and cultural practices. Colonial settlement in Australia impacted, threatened, and de-valued the practices of Aboriginal and Torres Strait Islander people, and these impacts are still evident today (Gee et al., 2020). Despite these human rights violations, Aboriginal families and communities have continued to provide their children with the quality of relational care that has nurtured generations prior, but that has been largely undocumented, placing critical knowledge systems at risk of being forgotten (Gee et al., 2020).

Australian Aboriginal Knowledge Systems provide sophisticated holistic relational care within kinship family and wider non-kin community collective networks (Gee et al., 2020). This collectivist approach to understanding relational health is often at odds with Western models of attachment that have typically focused on the relationship between parent (mostly the mother) and child, with limited consideration of the impact of the wider kin and non-kin social ecology (Ainsworth, 1979, 1985; Bowlby, 1969). In this way, Western approaches often miss the multi-dimensional relational concept of caring that extends well beyond immediate parental carers to carers at all levels of society, including care that comes from connection to Country (Yeo, 2003).

Furthermore, in Western models of attachment, little attention has been given to the much broader intergenerational context within which ways of relating are developed, maintained, and stably transmitted across generations. Yet intergenerational thinking is fundamental to Australian Aboriginal ancestral epistemology that sees ‘ways of knowing, being and doing in the world’ exchanged within and between generations, guiding each new generation in the process (Lohoar et al., 2014).

Prior to colonisation, Aboriginal women’s childbirth practices were relationally supported not only within family networks but also within the broader kin and non-kin social networks of Australian Aboriginal community (Marriott et al., 2019). Aboriginal mothers care for their children within a complex matriarchal system where the mother-baby dyad is supported by multiple ongoing inter-connections and attachments with other women within their eco-systems, and Aboriginal children are nurtured with extensive roles and obligatory relational care systems within multiple ecologies of their life (Marriott et al., 2019). This multi-faceted relational attachment system continues across adolescence and into young adult life, providing substantial protection and guidance that encourages shared responsibility for mothers with other matriarchal family, kinship, and community members (Marriott et al., 2019). From an Aboriginal viewpoint, Aboriginal mothers and offspring have substantial support and access to resources that provide multiple support mechanisms that nurture both the mother and child in their ecology which collectively supports the child.

Strong intergenerational networks were profoundly disrupted by colonisation, and the legacies of intergenerational relational trauma post colonisation are still present today in the lives of Aboriginal families and communities. From colonisation in the early 1800s, Aboriginal people were removed from land, had their family systems dismantled, were interned to slavery, and introduced to toxic substances and the ‘European way of living’. Genocides and policies in Australia such as *Forced child removal* and *Stolen Generations* dismantled and disintegrated Aboriginal family systems, further creating a fear-based discourse that ignored and under-valued Aboriginal ways of raising children. Their long-lasting impacts continue today. For example, the *Bringing Them Home* report *(1997)* was a national inquiry into the separation of Aboriginal and Torres Strait Islander children from their families. It provided evidence for Aboriginal and Torres Strait Islander victims of forced removal which further described the impact on their life both socially and emotionally and identified the intergenerational impacts that continue to maintain and dismantle Aboriginal child rearing practice in Australia (The Human Rights and Equal Opportunity Commission, 1997).

These systematic and progressive violations of the rights of Australian Aboriginal people have resulted in a cascade of unmet needs, from material deprivation to loss of security, to a disrupted sense of belonging, to disconnection from place in society and loss of autonomy and freedom to live the life Australian Aboriginal people value (Zubrick et al., 2014). As a direct consequence, Aboriginal people experience the highest rates of chronic disease; unacceptable, alarmingly high rates of child removal; extremely high incarceration rates; and the highest rates of suicide globally (Funston, 2016). Mainstream health care and social welfare policies continue to perpetuate this by ignoring the presence of intergenerational impacts of colonial settlement on the relational wellbeing of Aboriginal families and communities (Hunter, 1991). Racism and the persistent discrimination against Aboriginal people continue to reinforce disadvantage, acting as a major health determinant that perpetuates and contributes to health and social inequality (Kairuz et al., 2021; Priest et al., 2024; Thurber et al., 2022).

**Fig. 1:**
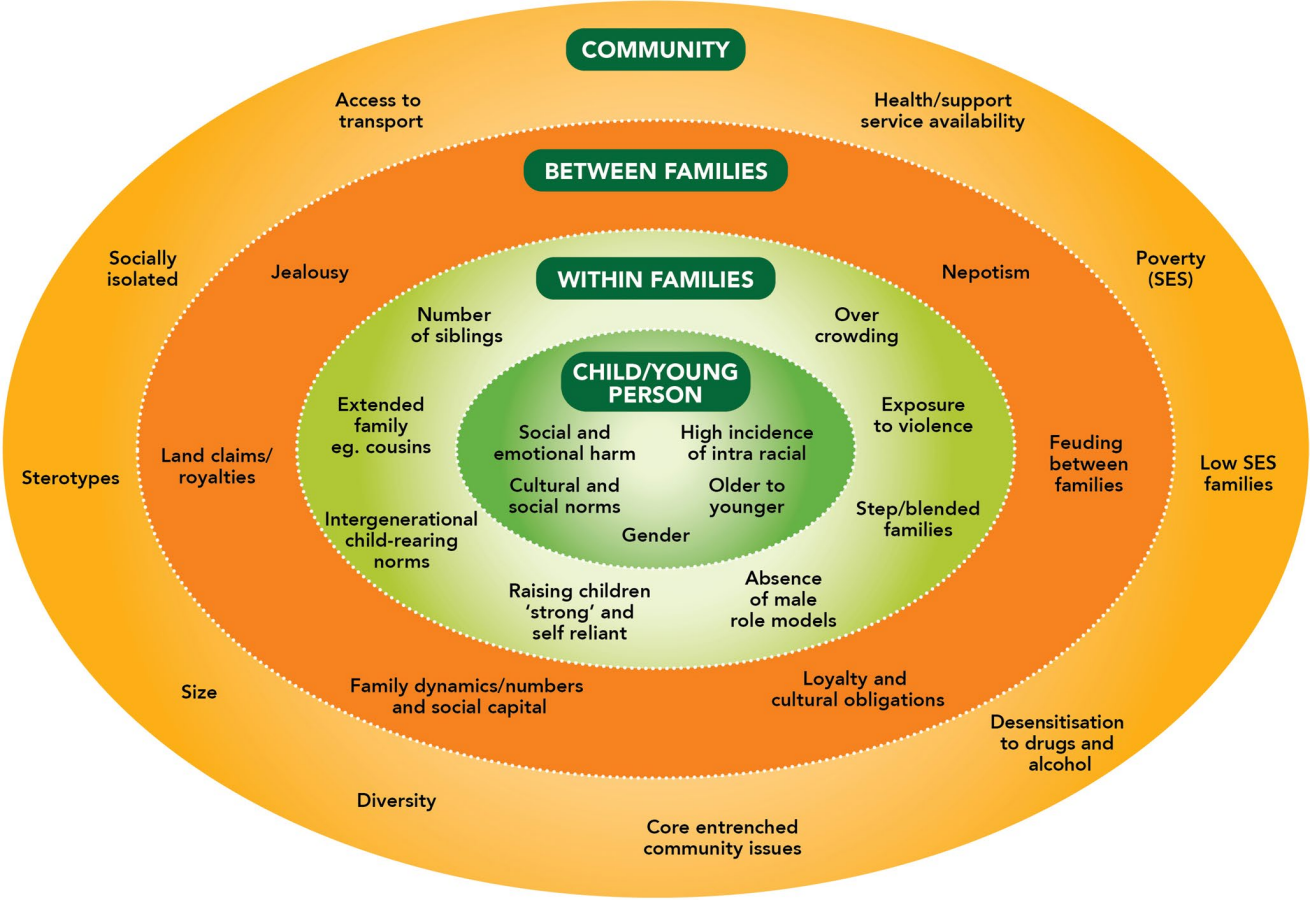
Coffin’s (2010) Social Ecological Model of bullying in an Aboriginal context

This present review of Australian Aboriginal Relational Knowledge Systems is founded on the contributions of three influential Australian Aboriginal academics. The first is an Aboriginal social ecological framework, proposed by Coffin (2010), derived from research contextualising bullying within Yamaji communities in Western Australia. This framework reconceptualises relational interactions through interspatial layers that incorporate culturally specific determinants often excluded from mainstream models (Fig. 1). Individuals are situated within a dynamic ecology of peers, family, and community, highlighting the reciprocal and evolving nature of relational experiences. It identifies both cultural and social determinants as integral to understanding the lived realities of Aboriginal children and emphasises that culture does not merely influence individuals—it actively shapes behaviours and relationships across ecological domains. In addition, Coffin’s model integrates intergenerational ancestral processes as the foundation of relational security, proposing that current relational strengths arise from historically secure foundations.

This generational transmission of wellbeing reflects Aboriginal epistemologies that embed holistic development across time and place. The model underscores the importance of culturally conceptualised assessment tools that reflect the multifaceted systems supporting Aboriginal children’s growth and daily interactions. Importantly, it positions Aboriginal culture not as a reactive solution but as a primary source of repair and nurturance, particularly through community and “on Country” experiences that restore ecological balance and promote sustained wellbeing.

**Fig. 2:**
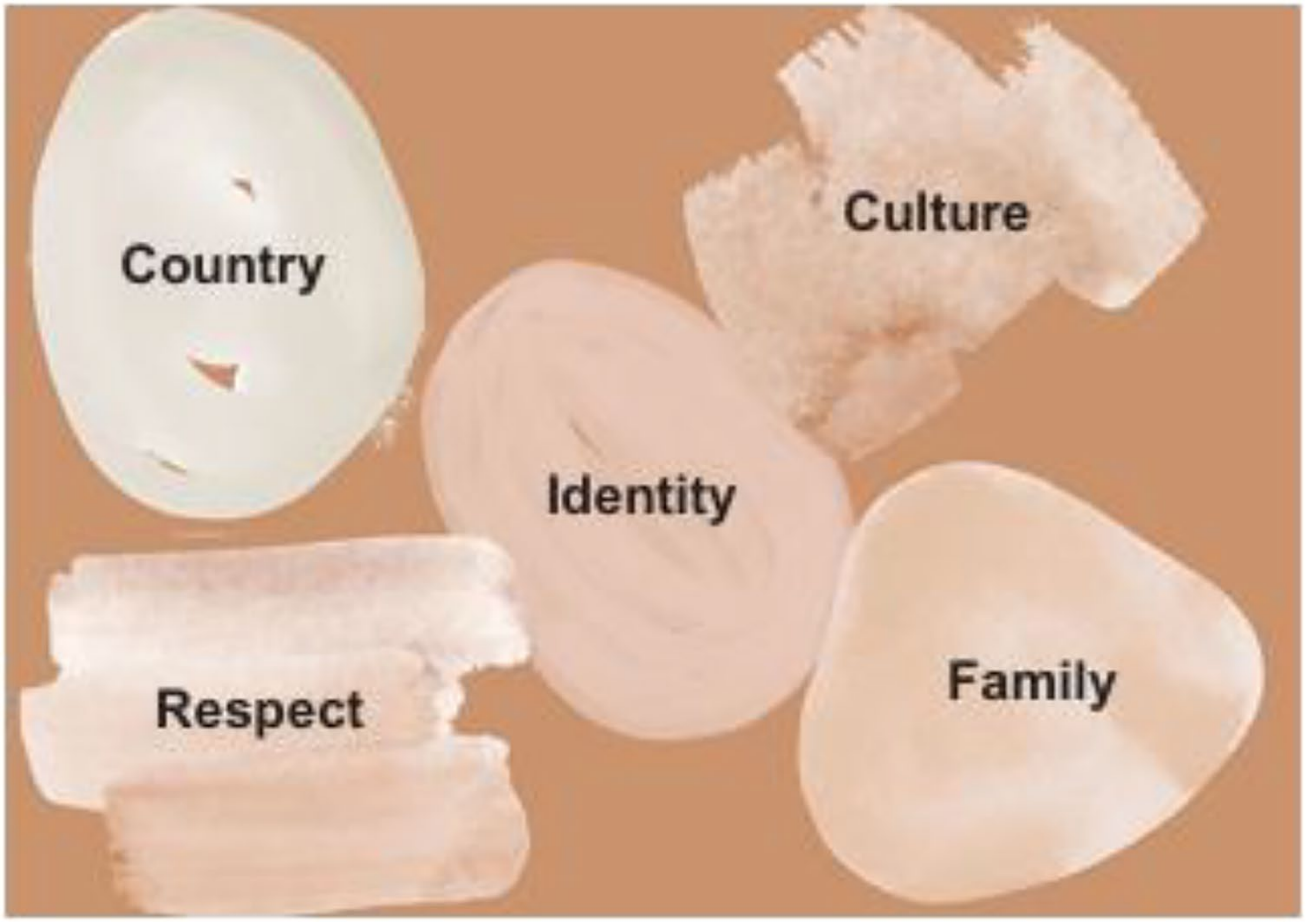
Wanganeen’s (2022) ‘First Nations Systems of Needs’ model

A second influence is from Australian Aboriginal researcher Professor Janneen Wanganeen who developed an aligned ‘First Nations Systems of Needs’ model (Fig. 2) based around five core needs: culture, Country, family, respect, and identity (Wanganeen, 2022). This framework de-constructs assessment modalities to allow exploration of Aboriginal people's experience through their own eyes, and on their own terms. Additionally, the framework embraces a narrative approach where Aboriginal community ethics overarches Wanganeen’s (2022) research and eliminates the inclusion of dominant Western centric frameworks. Wanganeen (2022) considered that the development of assessments for Aboriginal people required engaging and approaching the dominant Western worldview and frameworks which created the deficit discourse to Aboriginal ways of life and values.

In both models children benefit from a diverse range of secure attachments that emerge within multiple systems and ecologies which collectively nurture Aboriginal children across kin and non-kin social networks, including connection to Country. Aboriginal relational concepts of care have long encompassed wider kin and non‑kin networks and uniquely organised to meet the fundamental needs of early life.

**Fig. 3:**
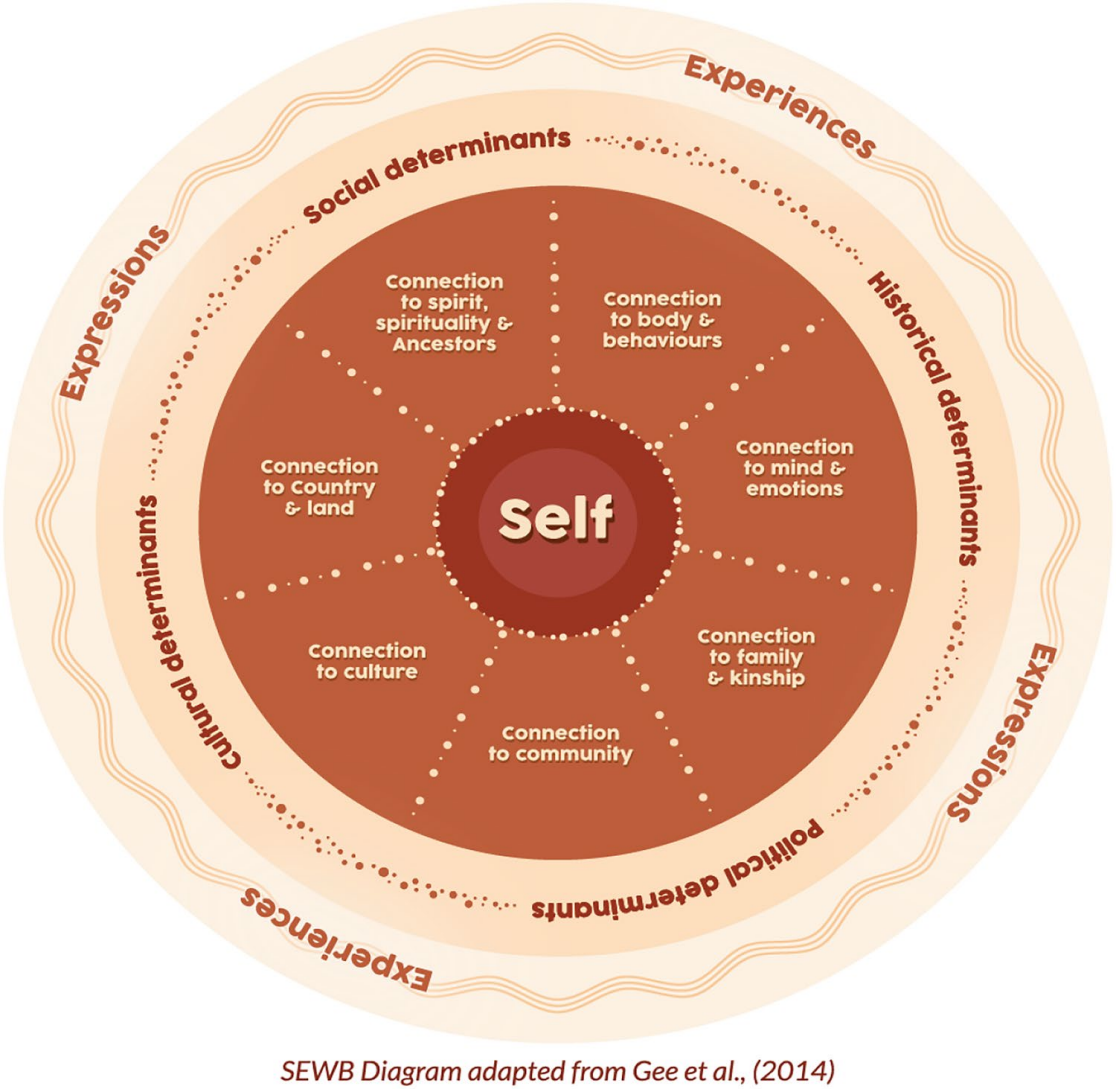
SEWB Diagram adapted from Gee et al. (2014)

A third leading Australian Aboriginal thinker in the area of relational wellbeing is Professor Graham Gee and colleagues (2014) who developed a framework of social and emotional health and wellbeing that provided a holistic view of Aboriginal life and included human, non-human, familial, cultural, and the environmental ecologies present in Aboriginal people's lives (Fig. 3). This concept of holistic health is not part of the discourse in mental health terminology or understood by mainstream health care services and providers in Australia. Gee et al. (2014) developed the social and emotional well-being (SEWB) framework that identifies Aboriginal and Torres Strait Islander health as ‘*a collectivist approach to an individual’s self-concept’.* The contextualisation of self being interconnected and embedded within family and community as a collective for Aboriginal people demonstrates the diversity of cultural groups and as individuals; that they have their own, unique experiences of social and emotional wellbeing (Gee et al., 2014). For children and young people an integration of all models could enhance a comprehensive understanding of Aboriginal relational health and wellbeing, where Aboriginal young people are understood within their own context.

One of the few conceptual parallels between Australian Aboriginal and Western thinking around the drivers of human development is with the work of Urie Bronfenbrenner (Bronfenbrenner, 1979, 2001, 2005). Bronfenbrenner’s bioecological framework aligns with Aboriginal thinking by proposing that developmental needs are met across multiple socio-ecological systems, defined within his model as the microsystems of family, peers, school, and community within which children interact with people, objects and symbols; the mesosystem of interactions between microsystems that improve the relational quality of microsystems; and broader exosystems and macrosystems of cultural norms and values, and the polices and institutions that support relationally healthy micro and mesosystems around the developing child. Aboriginal frameworks extend this model by also considering care for the next generation as fundamentally linked to both Country and spirituality. Bronfenbrenner’s work has been profoundly influential, yet it has rarely been accompanied by corresponding intervention frameworks that engage with the broader ecological determinants shaping child–carer relationships. This absence likely reflects the substantial complexity and practical challenges involved in designing and implementing interventions across multiple, interacting systems.

In our review, we reference all three Australian Aboriginal perspectives to conceptualise the relational context of early child development. We recognise the centrality of ecological thinking in Australian Aboriginal Relational Knowledge Systems. Importantly, Australian Aboriginal perspectives contextualise early relational health within an intergenerational timeframe, understanding that secure relational foundations in one generation emerge from secure relational foundations in prior generations.

In this way, Australian Aboriginal approaches advocate for promoting relational health not only in early childhood, but also in adolescence and young adulthood and on transition to parenting next generation offspring. Australian Aboriginal perspectives encourage deeper thinking; thinkiHere we aim to synthesise academic literature across Australia as it relates to the evidence of relational knowledge systems and cultural practices of Australian Aboriginal people. Additionally, this scoping review aims to identify and determine the constructs that promote relational health and wellbeing for Aboriginal children from early childhood to young adulthood. The decolonisation and privileging of Aboriginal knowledge and belief systems throughout this scoping review engages a focus on Aboriginal authored articles only. This prioritises Aboriginal research methodologies and promotes research rigour which challenge Western societal child development literature and methodologies that typically dominate, habituate, and continue to misinterpret Aboriginal worldviews (Dudgeon et al., 2024). Our review questions are:

1. What relational knowledge systems and cultural practices, as they relate to children and young people, are in Australian Aboriginal-authored literature?
2. What constructs are identified as promoting relational health and wellbeing for Australian Aboriginal children and young people?
3. What gaps exist in the literature regarding relational health and wellbeing for Australian Aboriginal children and young people?

ng that respects ancestry and the intergenerational embedding of health and wellbeing within and across generations.

### Positionality Statement of Australian Aboriginal Authors

Shannon McNeair is a Malgana woman from Shark Bay Western Australia, a Psychologist and Indigenous researcher with Deakin University and Murdoch University. Her area of research is focused on relational health and well-being of Aboriginal young people.

Professor Catherine Chamberlain is a Trawlwoolway woman from the North East Coast of Tasmania. She is a midwife and leads research to improve care for Aboriginal and Torres Strait Islander parents experiencing complex trauma.

Professor Juli Coffin is a Nyangumarta woman from the Pilbara in Western Australia. She is a leading Indigenous researcher in Australia and is the founder and lead researcher of the Yawardani Jan-ga research project and Equine Assisted Learning program based in the Kimberley.

## Methods

Since this study aimed to explore the breadth and depth of Australian Aboriginal-led research on Aboriginal practices for raising relationally healthy children and young people, a scoping review was selected as the preferred methodology. The review was conducted in accordance with the Joanna Briggs Institute (JBI) approach to scoping reviews (Peters et al., 2021) and follows the Preferred Reporting Items of Systematic Reviews and Meta-Analyses extension for Scoping Reviews (PRISMA-ScR) reporting guideline (Tricco et al., 2018).

A narrative synthesis of the literature was undertaken where Aboriginal authored articles were reviewed, and themes and constructs were identified using a thematic analysis approach of collaboration and participatory appraisal. Aboriginal researchers examined each article and engaged an Aboriginal participatory action research approach to review, identify, agree, and confirm themes and constructs (Dudgeon et al., 2020). Common and repeated relational health clustered across three main identified themes.

Each Aboriginal researcher utilised their personal lived experience, and community and cultural knowledge to collaborate on themes and constructs. This aligns with previous positionality statements where the inclusion of Aboriginal researchers lived experience, social, and political views, and beliefs, privileges Aboriginal knowledge and expertise and further attempts to decolonise research methodologies and processes (Dudgeon et al., 2024).

### Search Strategy

The search strategy was developed in consultation with a research librarian. Electronic databases (MEDLINE COMPLETE [EBSCOhost], PsycINFO [EBSCOhost], Embase, SocINDEX [EBSCOhost] and CINAHL [EBSCOhost] were searched in March 2024. Search terms combined 2 concepts: (1) Child and family relational ecology and (2) Australian Indigenous terms. The full electronic search strategy is provided in Online Resource 1. Searches were limited to human populations, English language and peer reviewed publications. No date limitations were applied.

To locate the Aboriginal-authored literature, relational mapping was applied which involved checking the reference list of each article, utilising Aboriginal community and research knowledge, as well as consultation with other Aboriginal community and knowledge systems. Relational mapping further enabled our scoping review to be inclusive of contemporary academic discourses, not just Western dominated ways of knowing, and added academic rigour.

### Information Sources

We considered all peer-reviewed quantitative, qualitative and mixed-methods study designs, irrespective of methodological approach. Non-peer reviewed evidence including reviews, editorial and opinion papers, conference proceedings, guidance documents, government documents, policy documents and books were not considered for inclusion. Theses and dissertations were considered peer reviewed by examiners and therefore considered for inclusion.

Underpinning the review is a strengths-based and decolonising approach which privileged literature with Aboriginal and Torres Strait Islander authors and Aboriginal and Torres Strait Islander research methodologies.

### Eligibility Criteria

Eligibility criteria were developed in line with the JBI recommended PCC framework (population, concept, and context; (Pollock et al., 2023).

*Population*: Studies with participants who self-identified as Australian Aboriginal or Torres Strait Islander were considered. The age of subjects for which relational health or relational principles were applied was limited to infants, children and young people prior to having offspring (approximately 0–25 years), although this was not always explicitly stated, and an inclusive approach was taken. No gender restrictions were applied. Studies examining the target population alongside other Indigenous populations from countries other than Australia were excluded.

*Concept***:** Studies describing or assessing cultural practices in Australian Aboriginal and Torres Strait Islander communities for promoting relational health from early childhood to young adulthood and into parenting the next generation. Early relational health refers to the quality of connections a child or young person develops with or observes between key figures in their relational ecology including caregivers, family and community members (kin, non-kin).

*Context***:** Sources published in international peer reviewed literature. The study focus could be on the general Indigenous population data or restricted to certain high-risk groups (e.g. first-time or young mothers, foster children, exposure to domestic violence).

### Selection of Sources of Evidence

All records from the search were uploaded into LitQuest (Fuller-Tyszkiewicz et al., 2025; Grbin et al., 2022) and duplicates were removed. Titles and abstracts were then independently double screened against eligibility criteria by two or more blinded study authors until a stop-point was indicated by LitQuest. As described more fully in Fuller-Tyszkiewicz et al. (2025), LItQuest uses supervised machine-learning models that learn from reviewers screening decisions to assist in excluding non-relevant studies. As title-abstract screening proceeds, the system updates its predictions and ranks remaining references by estimated relevance. LItQuest applies a pre-determined stopping rule: screening continues until reviewers encounters 40 consecutive non-relevant articles and all remaining and all remaining unscreened references have predicted relevance scores below 0.5. When these criteria are met, LitQuest prompts users to conclude screening on the basis that additional relevant studies are unlikely to remain. Screening conflicts were resolved by discussion to reach consensus.

At the full text screening stage, double screening was carried out on 47% of all articles, following which an inter-rater check was performed on 20 articles to assess the feasibility of moving to a single screening process. Inter rater reliability was found to be 90% and the remaining articles were single screened, with regular discussion between independent screeners regarding any uncertainty of an article meeting any aspect of the inclusion criteria. The reasons for exclusion at full text review were identified in the PRISMA flowchart of articles screened for inclusion in the scoping review. See Table 1 for inclusion and exclusion criteria for full-text review.

**Table 1:** Inclusion and exclusion criteria for full-text review

Inclusion	Exclusion
Written in *English*	Not written in English
*Human* subjects	Non-human subjects
Academic literature (peer-reviewed journal articles and dissertations)	Conference abstracts, editorials, opinion pieces, letters, study protocols, commentaries, book reviews
*Australian First Nations/Indigenous* subjects, including Aboriginal and Torres Strait Islander peoples	*Non-Australian First Nations/Indigenous* subjects
Study type: Quantitative or qualitative studies, mixed-methods, ethnographic, observational, intervention studies (including RCTs), and case studies	None
Examines the *relational health of infants, children, adolescents and/or young adults prior to child rearing* (from conception to ~ 25 years old), as reflected in the search syntax. This may include: • Infant/child/foetal attachment, bonding, relationships or interactions with any main caregiver/s • Any form of sensitivity, involvement, interactions, or investment from a main caregiver • Prenatal and birthing practices including accessing services and ‘birthing on Country’ • Any form of ‘parenting’ or nurturing that the child may be exposed to, e.g., parenting styles, parenting quality, child maltreatment • Parental or family relationships e.g. conflict, cohesion, interactions or functioning • Quality or functioning of peer relationships, including bullying • Quality or functioning of intimate partner relationships, including violence, abuse and sexual consent	Does not examine any aspect of relational health or examines relational health of adults after having children
At least one author declared in article as Australian Indigenous	No Australian Indigenous authorship declared

### Data Extraction

The data extraction form was pilot tested on five articles to assess reliability and consistency of the extraction as well appropriateness and usability of the form. Following pilot testing, data was extracted from included studies by one of four reviewers, with 10% cross-checked by another. The data extraction form included (1) general study, and sample characteristics, (2) methodology used, (3) relational health construct discussed (identified by non-Aboriginal authors and linked to the topics listed in the inclusion criteria), and (4) relational health construct identified by Aboriginal author (SM).

### Data Collation and Analysis

The process of data collation and analysis included elements of participatory action research such as participatory appraisal, rapid appraisal, participatory learning and action, and collaborative enquiry. For evaluation of the extracted content, the study-level constructs were compared across papers through a collaborative and participatory thematic analysis process. Repeated and conceptually overlapping constructs were grouped, refined, and discussed iteratively until agreement was reached. Information pertaining to the review questions is presented as narrative text to contextualise findings and knowledge gaps. Aboriginal participatory action research methods were utilised within the Aboriginal research team to synthesise the narrative data collaboratively and concur on findings and knowledge gaps (Dudgeon et al., 2020).

## Results

### Selection of Sources of Evidence

After deduplication, the search yielded 5141 records. Manual screening of titles and abstracts was performed on 2,739 reports (53.3%), excluding 2,488 of them. LitQuest excluded the remaining 2,402 reports.

**Fig. 4:**
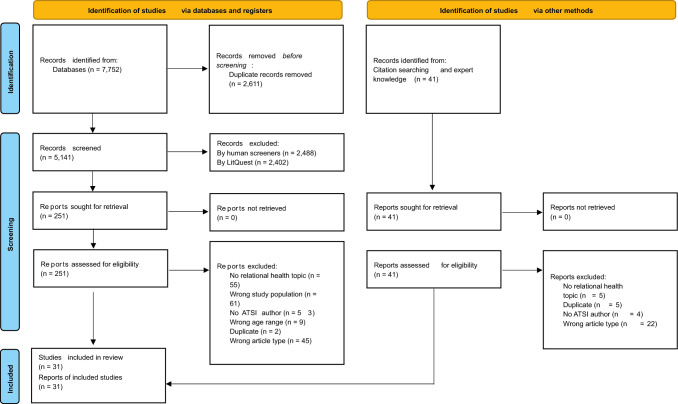
PRISMA flow diagram of the study selection process

Full-text screening was conducted on 251 reports, resulting in 26 inclusions. Additionally, five reports were identified through citation searching and expert knowledge. Each report corresponds to a unique study. Therefore, the findings are based on data extracted from 31 reports of 31 unique studies. The PRISMA chart in Fig. 4 illustrates the flow of studies at each review stage . Study details of the 31 included studies are provided in Table 2.

**Table 2:** Details of included studies

Study ID	Methodology	Brief description of participants	Indigenous status of participants^a^	Community/location	Sample size	Participant age	Relational health construct examined	Relational health construct identified by Aboriginal author (SM)
Armstrong et al. (2022**)**	Qualitative (ethnography)	Yolŋu children and their family members Community members	Aboriginal (Yolŋu)	North-East Arnhem Land (NT)	44	Children: Range: 2 months—2 years. Followed longitudinally for 7 years Family members: NS Community members: Range: 18 –78 years	Parenting practices Family relationships Kinship	Kinship Intergenerational impact Family responsibilities Affiliation with community
Atkinson and Swain (1999**)**	Qualitative (interviews)	Koorie women working in community organisations or Koorie-identified positions in mainstream educational institutions who were recognised as having a maternal role within the Koorie community	Aboriginal (Koorie)	VIC	11	Range: 25 -71 years	Parental and family relationships Intimate partner relationships Bullying (due to racism) Kinship	Identity & culture Kinship systems Connection to Country SEWB Self determination Intergenerational trauma Ancient wisdom
Bailey and Clark (2024**)**	Qualitative (yarning)	Mental health professionals who (1) identified as Aboriginal, or (2) had extensive knowledge and experience working with Aboriginal families on bonding and attachment	Aboriginal and non-Aboriginal	Kaurna lands (SA)	8	NS	Bonding and attachment Parenting Kinship and family relationships	Intergenerational trauma Culture is central to identity formation
Beaufils (2023**)**	Qualitative (interviews)	Group 1: Young First Nations people (18–30 years) with involvement in the NSW out-of-home care (OOHC) system in the previous 10 years Group 2: People currently involved in the OOHC system (e.g., parents, other family members, carers and guardians, community members, key OOHC departmental and agency staff)	First Nations and non-Indigenous	11 communities in metropolitan, regional, and remote areas of NSW	37	Group 1: Range: 18–30 years Group 2: NS	Kinship and care of children raised in out-of-home care (OOHC) system	Kinship Culture regulating relationships Self determination Culture & connection
Byers et al. (2012**)**	Qualitative (ethnography)	Families with children aged < 5 years	Aboriginal	Remote community in Central Australia (NT)	8	Range: < 5 years	Parenting styles Relationships with main caregivers Caregiver involvement and interactions Family relationships	Communal social culture Staying strong Autonomous parenting
Canuto et al. (2019**)**	Qualitative (yarning)	Men who identified as ATSI and were parents or provided nurturing and/or caring roles and responsibilities	ATSI	Four locations in SA: Coober Pedy, Yalata, Port Lincoln, and metropolitan Adelaide	46	Range: 18 + years	Parenting roles and responsibilities Family relationships	Culture & community connectors Respect systems Family & kinship Responsibilities
Chamberlain et al. (2021**)**	Qualitative (interviews and discussion groups)	Parents, mostly mothers, recruited through community networks and perinatal care services. Participants were currently pregnant or had a child aged < 2 years	ATSI	Three locations: Melbourne (VIC), Alice Springs (NT), and Adelaide (SA)	17	Mean = 29 years	Parental and family relationships	SEWB Connectedness Kinship Family systems
Eades et al. (2020**)**	Qualitative (interviews)	Women with chronic disease (all had diabetes, some also had chronic kidney disease or cardiovascular disease)	Aboriginal	WA, QLD, Central Australia (NT)	72	Mean = 53 years Range: 26–80 years	Family caring roles and responsibilities	Intergenerational trauma Family as strength Country Cultural identity
Funston (2016**)**	Qualitative (yarning circles)	Aboriginal Communities Matter Advisory Group (ACMAG)	ATSI	NSW	NS	NS	Parental or family relationships	Intergenerational trauma Systemic impacts of racism and discrimination
Gair et al. (2019**)**	Qualitative (interviews and focus groups)	Primarily grandparent carers for grandchildren in child protection system, as well as foster carers (non-relatives), parents and workers in child protection support roles	ATSI and non-ATSI	QLD, WA and VIC	77	Range: 36–66 + years	Parental or family relationships Intimate partner relationships	Kinship systems Protectiveness of family
Gee et al. (2020**)**	Qualitative (yarning circles)	Parents who had experienced trauma and healing	Aboriginal	Melbourne (VIC)	6	Range: 35–55 years	Parenting practices	Culture, community & history
Grace et al. (2016**)**	Mixed methods (survey, interviews)	Aboriginal families and staff in Parents as Teachers (PAT) home visiting programme	Aboriginal	Remote NSW	29	Mean = 17.7 months Range: 1—36 months	Parenting	Community effects Parenting as capacity building
Islam et al., (2022**)**	Quantitative (survey)	First Nations adolescents	ATSI	11 Indigenous communities in urban, regional and remote settings across Australia	365	Mean = 14.04 years (SD = 0.45)	Family cohesion	Protective factors: Strong family cohesion, cultural sensitivities in education programs, community leadership
Kruske et al. (2012**)**	Qualitative (ethnography)	Mother-infant pairs and others involved in the child's rearing—typically the infant's father, grandmothers and aunties	Aboriginal	Remote communities in Northern Australia	15	Range: Birth—1 year	Family relationships	Child-led parenting style Behaviourally & developmentally Collectivist culture Relationships
Lilley et al. (2023**)**	Qualitative (interviews)	Women in a caring role for an autistic child	ATSI	Across Australia	11	Range: 2–22 years	Parenting Family practices	Protectiveness Respect Helping others/families in community
Lowell et al. (2015**)**	Qualitative (interviews)	Past and present workers on 'Strong Women, Strong Babies, Strong Culture' Program, staff of local organizations, community members, and Department of Health staff	Aboriginal and non-Aboriginal	Five remote communities in NT	76	NS	Childbirth Prenatal and antenatal care for young mothers	Traditional health knowledge & practice Cultural knowledge and practice Respect
Lyall et al. (2023**)**	Qualitative (yarning)	Community members diverse in age and gender	ATSI	Yuggara Country—Inala, Brisbane (QLD)	10	Range: Mid-20 s to 70 s (years)	Child raising/parenting, including pregnancy care Community connectedness	Indigenous ways of intergenerational learning
Malin et al. (1996**)**	Qualitative (ethnography)	Compilations of data from two Nunga and two Anglo families	Aboriginal and non-Aboriginal	Aboriginal families were from Adelaide (SA). Location of Anglo families NS	4	NS	Parenting Family relationships Kinship	Independent, self-reliant & self-regulating
Marriott et al. (2019**)**	Qualitative (yarning)	Aboriginal birthing women, senior women and elder women. Four generations were represented- grandmothers, mothers, daughters, and granddaughters	Aboriginal	Wajuk—WA	74	Range: Conception—birth	Birthing practices	Intergenerational knowledge supporting cultural security Intergenerational sharing
Massi et al. (2023**)**	Qualitative (yarning – individual or circles)	First time mothers/first opportunity to parent, having a First Nations baby. Participants had enrolled, declined enrolment, dropped out of, or graduated from the Australian Nurse Family Partnership Program	ATSI	Meanjin, Brisbane (QLD)	29	NS	Parenting practices and support	Relationships and connection to culture & identity Personal growth and transformation (strength)
McLachlan et al. (2022**)**	Mixed methods (booking schedules, routine data sets and caseload midwives’ records)	Pregnant women having a First Nations baby (70% ATSI) and healthcare providers (including midwives, social workers, managers and Aboriginal Hospital Liaison Officers)	ATSI	Three major metropolitan maternity services in Melbourne, VIC	1,040	NS	Prenatal and birthing practices including accessing services	Culturally responsive support
McMahon (2017**)**	Qualitative (Indigenous relational research, ‘Banburra’)	Indigenous community leaders	ATSI	10 communities invited to participate: Yorta Yorta in VIC; Erub Island in Torres Strait Islands; Wergaia in VIC; Yolngu in Arnhem Land; Central Australia; Palm Island; Nunga in SA; Burarra in NT; Noonuccal in QLD; and Anangu and Yapa in NT	5	NS	Parenting Family relationships Kinship	Intergenerational trauma Culture is central to identity formation
Miller et al. (2020**)**	Qualitative (open text survey responses)	Aboriginal children and their carers (carers aged 16 years + , not all carers identified as Aboriginal)	Aboriginal and non-Aboriginal	Regional and urban NSW	425	Mean = 35 years Range: 18–66 years	Family relationships	SEWB Active community lifestyle Engaging in community & community lifestyle Strong culture
Parker et al. (2014**)**	Mixed methods (survey – quantitative and qualitative data)	ATSI women who had a live singleton birth between 1 July 2011 and 1 July 2012	ATSI	QLD	187	Age distribution: 16–24 years (52.9%), 25–29 years (23%), 30–39 years (23%), > 40 years (1.1%)	Family relationships	Respect Cultural practices
Ponnapalli et al. (2023**)**	Qualitative (focus groups and interviews)	ATSI parents/carers of at least one pre-teen living in the home	ATSI	an Indigenous community in South-East QLD	17	Range: < 13 years	Parenting	Culture, Country & spirituality SEWB
Reilly and Rees (2018**)**	Qualitative (interviews, yarning groups)	Expert sample of stakeholders with specific knowledge of men’s parenting: Group 1: 25 ATSI stakeholders (16 males, 9 females) and Group 2: 6 non-ATSI stakeholders (2 males, 4 females)	ATSI and non-ATSI	3 remote communities in the Lower Gulf of Carpentaria, QLD	25	Group 1: Range: 25–75 years Group 2: Range: 40–60 years	Parenting	Traditional community roles
Rohit et al. (2021**)**	Qualitative (interviews)	Aboriginal parents with children between the ages of 2–5 years, service providers with key knowledge of child health and nutrition from the same geographic location	Aboriginal and non-Aboriginal	6 locations in NT	59	Parents: Mean = 30 ± 7 years Children: Mean = 3 ± 1 year Service providers: NS	Parenting style (related to child feeding practices)	Valuing child autonomy Sharing as cultural practice in parenting Collectivist approach
Rynne et al. (2023**)**	Quantitative	Parents of JRL (Junior Rugby League) players aged 8 to 16 years	Aboriginal and non-Aboriginal	QLD and NSW	117	Parents range: 27–66 years Children range: 8–16 years	Parenting Community connectedness	Kinship Affiliation with community Family participation Active community lifestyle
Sullivan et al. (2022**)**	Qualitative (interviews)	Young Indigenous LGBTIQSB + (Lesbian, Gay, Bisexual, Trans Intersex, Queer, Sistergirl, Brotherboy +) people who reported having ATSI mothers	ATSI	Urban and regional areas of NSW	9	Range: 14–25 years	Mother–child relationship	Unconditional love within and external to community
Turnbull‐Roberts et al. (2022)	Qualitative (submissions to government inquiry)	Submissions to the 2018 Australian Senate Parliamentary Inquiry into Adoption Reform from Aboriginal community-controlled organizations	ATSI	National and state-based organisations	NS	NS	Family relationships Attachment Child maltreatment	Self determination Autonomy Intergenerational trauma
Vujcich et al. (2018**)**	Qualitative (focus groups and interviews)	Aboriginal carers of children or adolescents	Aboriginal	Rural and urban WA	81	Range: 5 months—40 + years	Family relationships—specifically talking about sexual health / relationships with their child	Culture empowering relationships

^a^ATSI = Aboriginal and Torres Strait Islander representation. Aboriginal = Aboriginal representation only

### Description of Included Studies

Included reports were published between 1996 and 2024, with most (29, 93.5%) published since 2011. The majority of studies used a qualitative design (26, 83.9%). Study participants included: children and young people (5 studies, 16.1%), their parents, carers and other family members (22 studies, 71%), community members, including Elders and community leaders (8 studies, 25.8%), or health, education or other professionals working in Aboriginal and/or Torres Strait Islander communities (10 studies, 32.2%). In eight studies (25.8%), two or more of these groups were consulted. Almost a half of the studies (15/31, 48.4%) identified intergenerational ancestral determinants contributing to the early relational health; confirming that secure relational foundations in prior generations support embedment in current and future generations. A quarter of studies (7 studies, 25.8%) reported on samples from multiple states or territories. Samples were drawn from all Australian states and territories except Tasmania and the Australian Capital Territory.

### Identified Constructs and Themes

#### Theme 1

##### Centrality of Kinship and Belonging within Collective Relational Systems

Kinship is a fundamental cultural element enabling belonging and relationality, extending beyond immediate blood relations, that provide extensive networks of social relationships which share a common commitment to protecting, nurturing and teaching children and young people (Chamberlain et al., 2021). In this way, Aboriginal communities rear children in a collective environment, sharing parenting responsibilities across and within the kinship system. Kinship is a fundamental, complex element of culture and a form of governance, encompassing relationships with family, community, culture, spirituality, Country, and all creation. Much of this complexity is lost in the institutional use of the word (as in ‘Kinship care’) (Beaufils, 2023; Ponnapalli et al., 2023; Sullivan et al., 2022).

Strong cultural belief systems for Aboriginal families emphasise hope and happiness with the birth of their offspring (Chamberlain et al., 2021). Babies bring new roles to mothers, as well as new hope to communities. Responsibilities of the community provide a rich resource and extension to the new role of the mother for her offspring and further imparts collective wisdom from different knowledge holders. Chamberlain et al. (2021) further suggests that the birth of a new baby creates a protective role and protective factor for a young mother and the opportunity for a woman to enter into the role of motherhood is tied to a strong cultural belief system where mothers are now positioned to receive privileged information from matriarchs and attain a high level of accountability to them (Chamberlain et al., 2021).

Knowledge exchange and transfer of responsibilities occur in new motherhood roles for Aboriginal women, which is a unique point of difference to the Western concept of a mother–child dyad of secure attachment. This difference in parenting distinguishes Aboriginal parenting from Western concepts of a dyadic relationship and considers a much more complex interactive ecology in the nurturing of offspring. Aboriginal ways of parenting their offspring teaches knowledge attainment from an early age and utilises an organised cultural belief system of care that nurtures baby and mother interchangeably throughout their life course (Chamberlain et al., 2021).

The collective responsibility of an Aboriginal community to undertake, determine, and allocate roles, and processes that nurture the mother–child relationship provides integral overarching support for the child’s human and individual development. Byers et al. (2012) descriptive study further highlighted that mainstream assessments of children did not include a collective community approach and reviewed children’s development in an individual context only. The cultural context of Aboriginal children is absent and not considered as an identified strength in the assessment of Aboriginal children’s well-being, which creates disparities and misconceptions in their individual presentation and outcome measures.

Kinship is described consistently throughout the literature as a construct that supports Aboriginal parents to collectively manage responsibilities and risks to their offspring (Ponnapalli et al., 2023). This kinship network of an Aboriginal child was identified by Ponnapalli et al. (2023) as a protective factor that continually mitigated risk in situations which otherwise would normally impact on individual parents negatively. For example, involving more community members in the collective parenting of their children incorporated increased supervision, increased leadership in community, and decreased need to engage punitive behaviour management techniques. Again, this significant difference in parenting within Aboriginal communities risks being misunderstood with Western societal parenting constructs, given that the parent–child relationship is defined as an individual relationship connection between one to two parents and child.

#### Theme 2

##### Centrality of Cultural Identity within Collectivist Relational Systems

The development of children and young people in Aboriginal culture occurs as a process of seamlessly integrating the individual into the vast web of culture, Country, and community, ensuring development of a clear sense of identity, belonging, and shared responsibility (Bailey & Clark, 2024; Beaufils, 2023; Lyall et al., 2023; McMahon, 2017). Developing a strong Indigenous identity is a high priority for families. Identity is established through cultural connections from conception. A strong cultural identity and engagement have been shown to strengthen resilience, build self-esteem, and foster pro-social coping mechanisms, which are essential for mental health and wellbeing (Armstrong et al., 2022; Byers et al., 2012; Chamberlain et al., 2021; Islam et al., 2022).

Bowlby’s (1969) attachment theory focuses on the parent–child dyad, particularly the mother–child relationship. Ainsworth (1979) incorporated secondary attachment figures such as fathers, but in a supplementary role to the primary mother–child dyad. Both theories were influenced by Western perceptions of the nuclear family which have consistently impacted collectivist ideas of ‘family’ negatively. Bailey and Clark (2024) stated that “W*estern attachment theory*” was unable to account and consider the “*naturally occurring differences in parenting and child rearing styles, behaviours, and environments”*. Bailey and Clark (2024) further stated that valuing interdependence, spirituality, community loyalty, traditional links to “Country” (or land) and group cohesion were present in an Aboriginal child's environment from an early age, but that these constructs were not broadly believed in mainstream development literature to assess attachment (Bailey & Clark, 2024).

Reliance on direct and observable interactions to measure secure attachment and human interactions presents additional barriers due to the limited literature supporting cultural methods of contextualising attachment for Aboriginal parents within a Western constructed assessment system (Lyall et al., 2023). In Western dominant attachment theory, direct observations form the basis of child assessment; however, the unconscious biases of clinicians contribute to misinterpretation of relationality and attachment between Aboriginal parents and their offspring. For example, Aboriginal remote communities are geographically situated in locations with limited access and resources. The obvious lack of resources and focus on materialistic possessions in Western society de-values Aboriginal remote communities. This maintains an observable misunderstanding, misrepresentation, and de-valuing of Aboriginal parenting and community life to the Western world (Lyall et al., 2023).

Lyall et al. (2023) further stated that resources for Aboriginal children were in the form of connections with their Aboriginal organisations as pillars of support that enhanced cultural connectivity within and between communities. This collective concept of resourcing contrasts with Western societal materialistic resourcefulness and maintains the stance that non-observable constructs to nurture the wellbeing of offspring exist (Lyall et al., 2023). This is a strength and a protective factor that creates a strong resource that is accessible to many. However, it is not observable and well-understood within Western society. To truly define the concept of “Country”, firstly requires relational wellbeing being to be understood as a relational extension of an environment attached with positive belief and meaning. This is a critical difference between Aboriginal relational care and Western concepts of care. Aboriginal relational care existed in the context of genocide and severe historical impacts which in turn evidence proof of strength in the generations of Aboriginal families in existence today.

Beaufils (2023) conducted research with Aboriginal children placed in out-of-home care and suggested that maintaining a connection to Country supported children in child placements to develop protective factors that were otherwise absent. Aboriginal children in out-of-home care placements with non-Aboriginal carers are a vulnerable cohort that particularly struggle with their cultural needs being met. Beaufils (2023) recommended that contemporary child protection policies should consult and engage with Aboriginal communities and stakeholders to support decision making on Aboriginal child placements. “Off Country” care also means that Aboriginal children do not have access to the complex layers of societal structures that support their nurturance and therefore lack access to the multiple parent roles they would have “on Country”. Additionally, the ability for an Aboriginal child to maintain their connection to Country and culture should be reviewed by Aboriginal families rather than the Western systems and policies that have elicited the child removal process (Beaufils, 2023).

The extension of the Aboriginal family system is inclusive of non-human entities and was discussed by McMahon (2017), Beaufils (2023), and Armstrong et al. (2022) as a more broadly changing definition of family. McMahon (2017) provided examples where First Nations people of the Anishnaabe Nation (Canadian Aboriginal peoples) included deer and turtle as clan members of their family system and within their extended family ecosystem. This is another example of the presence of a plethora of resources and a belief system that is not considered in mainstream Western society where emphasis is placed on relationships between human entities only. Relational well-being for Aboriginal children and young people could therefore be interpreted as interactive within a collective eco-system; seen or unseen, present and absent, biological and non-biological, human and non-human. Interchangeable processes between systems within an Aboriginal child’s ecology can be accessed at different ages and stages of development and considered contextually and in separation to the dominating Western child development frameworks and theories that de-value Aboriginal ways of living.

#### Theme 3

##### Centrality of Autonony and Self Reliance within Collectivist Relational Systems

Australian Aboriginal families highly value autonomy in their children, encouraging the development of independence and self-reliance from an early age within a close and nurturing environment (Byers et al., 2012; McMahon, 2017; Rohit et al., 2021). In this way, autonomy is enabled by strong family, kin and community relational networks that support children’s exploration and learning. In Aboriginal culture, young people are given opportunity for naturally occurring learning that is overseen by parents, particularly mothers and other female kin and non-kinship networks, who monitor more from a distance, only intervening when necessary, with the explicit aim of creating learning opportunities. Aboriginal children are cared for within a collective system where they have the freedom to live and move around their community and be more autonomous (Byers et al., 2012; McMahon, 2017; Rohit et al., 2021).

Males also play key roles in supporting autonomy and self-reliance, within kinship networks that may or may not involve the presence of the biological father (Canuto et al., 2019; Reilly & Rees, 2018). The male role tends to be more about protecting families, teaching survival skills (hunting and gathering), and supporting young males to build the skills to protect their families and communities in time. These processes do not often take the form of direct teaching; rather children and young people are invited to participate in communal opportunities and by doing so learn by observation and participation in ceremony and practices to embed cultural and familial roles.

In both male and female parental carer roles, children and young people actively participate in the processes of everyday life and learn by default by being there. However, lack of more direct parental intervention can be mistakenly viewed from the dominant culture as sub-optimal, which reflects a critical misunderstanding of the more indirect processes at play in Aboriginal parenting. Parenting in Aboriginal culture is less a direct, instructive process, and more of an emergent and observational learning process (Byers et al., 2012).

Byers et al. (2012) conducted a descriptive study which showed that Aboriginal children who were provided praise and encouragement from their parents, as well as kinship and community members in their community eco-system within their kin and non-kin relational networks demonstrated a level of physical and emotional maturity beyond their age (Byers et al., 2012). Byers et al. (2012) also found that Aboriginal offspring develop their independence and autonomy from an early age and therefore are valued resources for their communities. Aboriginal parents in this study encouraged their offspring to become self-reliant much earlier which further encouraged social maturity and autonomy. However, this can also be misinterpreted often, where perceptions of parentification are cast on young Aboriginal children and not understood in context.

Kruske et al. (2012) likewise showed that Aboriginal children's relational wellbeing was further underpinned by a cultural belief system that cognitively scaffolds implicit cultural continuity and self-governance. Cultural continuity and self-governance provide Aboriginal children with the ability to safely manage themselves in context at appropriate ages and stages. Cultural connectedness to family and community provides strong protective factors as Aboriginal children progress across their life course; however, measurement of cultural connectedness has remained dependent on Western assessment methods that don't capture the strengths of Aboriginal relational wellbeing.

Kruske et al. (2012) and Rohit et al. (2021) state that risk factors increase for Aboriginal young people in the absence of cultural connectedness, therefore limiting the identification of risk, and furthermore maintaining the ability to intervene appropriately. Aboriginal family eco-systems contain resources and strong protective factors that are not directly observable or understood in context outside of Aboriginal culture.

## Discussion

In this scoping review of the literature on Australian Aboriginal Relational Knowledge Systems we identify a range of distinguishing features of Australian *Aboriginal ways of knowing, being and doing*—individual, community and Country—that nurture the health and development of Australian Aboriginal children and young people from the earliest years of life. Through thematic analysis, within an Aboriginal participatory research framework, we identified three major conceptual themes, across the 31 papers included, which centre around the importance of Kinship and Belonging, Cultural Identity, and Autonomy and Self-Reliance showing that Aboriginal child-rearing is grounded in kinship as a culturally governed system of collective care in which extended relational networks (including family, community, spirituality and Country) share responsibility for protecting, nurturing and teaching children; that children’s wellbeing is inseparable from cultural identity, belonging and connection to Country, with these collective foundations fostering resilience and development; and that autonomy and self-reliance are intentionally cultivated through everyday observational and experiential learning under distributed supervision within kin and community networks.

The frameworks developed by Gee, Wanganeen, and Coffin offer a foundational lens for understanding Aboriginal relational contexts and have significantly advanced recognition of the strengths and wellbeing needs of Aboriginal children and young people across diverse communities. They initiate a conceptualisation of Aboriginal relationality grounded in Aboriginal worldviews rather than framed in opposition to Western models, supporting a more culturally anchored understanding of relationality as it is expressed across many Aboriginal communities in Australia.

A continuing barrier to preserving Aboriginal Relational Knowledge is the absence of culturally valid assessment tools and policy frameworks. Current child welfare and developmental assessment systems often overlook relational wellbeing, cultural connectedness, and ecological caregiving, leading to misinterpretation and inappropriate interventions. To address these gaps, practice and policy must shift toward culturally grounded, strengths-based assessment tools that reflect Aboriginal kinship structures and collective parenting norms. This requires co-designed assessment tools, culturally safe service delivery, and policy reforms that reframe autonomy and collectivism as protective rather than problematic, ensuring that Aboriginal families are supported in ways that include and honour their cultural integrity and promote lifelong wellbeing.

### Limitations

Firstly, Indigenous authored literature is limited and minimal. We adopted a stringent approach to determining Australian Indigenous authorship, only including articles where Indigenous status of one or more authors was declared. It is possible that some articles authored by Australian Indigenous authors were excluded because Indigenous identity was not explicitly stated. Some Indigenous researchers may choose not to disclose their identity for personal, cultural or privacy reasons and conversely, author declarations may not necessarily reflect the depth of Indigenous involvement in the research. While our aim was to highlight the voices and perspectives of Indigenous scholars by focussing on research led or co-led by Indigenous authors, this approach may have introduced bias, leading to the findings not fully representing the breadth of Indigenous scholarship in this field. Although Indigenous research evidence is increasing, it still remains disproportionate to the need.

Secondly, the literature used with Indigenous authorship again was minimal compared to wider theories and Western societal constructs already evidenced thus creating limitations. The representativeness of the diversity within Aboriginal populations in Australia could therefore be questionable to the wider community. Indigenous research methodologies and cultural governance groups can address these disparities for Aboriginal researchers where cultural guidance and direction can support inclusion and identification of individual constructs representative of the diversity within a diverse population of a specific Aboriginal community. In addition, the Torres Strait Islander literature was further limited given that majority of the authorship identified as Aboriginal only.

Thirdly, given our focus on Australian Aboriginal knowledge systems, we did not undertake a systematic cross-cultural comparison with other Indigenous peoples internationally, or with collectivist cultural frameworks more broadly. Future research could usefully examine points of convergence and divergence, including how relational responsibility is variously constituted in kinship- and community-based systems compared with dyadic parent–child models.

The current review also highlights the increased need to develop assessment measures that are culturally valid and reliable as well as measure the individual constructs that directly, validly, and reliably assess the relational well-being of Aboriginal children and young people. Currently, assessment is still limited to Western standard child development assessment tools, and only a small proportion of these assessments have been re-constructed. Most assessments utilised are redeveloped cultural adaptations of mainstream assessments and therefore maintain the stance of deficit discourse of Aboriginal children and young people being considerably disadvantaged in all aspects of their life and development. The findings from this review will complement and support community-led, culturally grounded ways of *knowing, being and doing* (e.g., yarning, participatory action research, community governance processes, and other Indigenous methodologies) for generating nuanced knowledge and guiding measure and intervention development.

### Actionable Insights

To preserve and strengthen Australian Aboriginal Relational Knowledge Systems we recommend the following for future cultural investment:

1. That opportunities to identify individual constructs that contribute to the relational wellbeing of Aboriginal children and young people should be conceptualised within an ecological framework that considers a wide range of kin and non-kin relationships, that could be captured from examination of programs currently promoting the relational health and wellbeing of Aboriginal children and young people.
2. That Aboriginal children and young people are understood within their own cultural context and identity that is held within large relational networks. Additionally, that the inclusion of other natural constructs in Aboriginal social ecologies, such as storylines held in totems, caring for Country, and animals as protectors and healers, are relational extensions of environment that children and young people grow up in, which are attached with positive belief and meaning.
3. That Australian Aboriginal parenting practices that support autonomy and self-reliance be better recognised, accepted, and understood as culturally valid, normal, and effective approaches to raising children and young people well.
4. That research prioritising *Aboriginal ways of knowing, being and doing* informs the development of culturally valid and reliable assessment tools for measuring relational wellbeing that can be used to gather the data needed to effect real change in policy and practice design, implementation, and evaluation. Such a process, which would also include the careful consideration of what can be adapted from existing tools, would contextualise information gathering as a *purposeful process* capable of supporting Aboriginal community led initiatives to promote strengths, develop and sustain initiatives, that empower communities to self-govern across all ecological systems (including schools, police, welfare services, and local government) that shape the development of Aboriginal children and young people.

## Conclusion

The relational wellbeing of Aboriginal children and young people is shaped within a highly interconnected ecosystem that encompasses multiple dimensions of human development, from individual experiences, to rich relational networks, to deep connections to the natural world. Aboriginal relational knowledge systems conceptualise Aboriginal families and communities within environmental, spiritual, and non-human systems as integral and interactive components of their everyday ecologies—within homes, across communities, and in individual lives. Evidencing Aboriginal relationality strengthens the development of interventions that align with and are responsive to *Aboriginal ways of knowing, being, and doing* and further enables and supports addressing the identified needs congruently with Aboriginal relational priorities and practices.

## Supplementary Information

Below is the link to the electronic supplementary material.

Supplementary Material

## Data Availability

No datasets were generated or analysed during the current study.
