# Supplementary material for: A Scoping Review of Australian Aboriginal Early Relational Health Knowledge Systems
Source: Clin Child Fam Psychol Rev. 2026 May 2;29(2):256–75. doi: 10.1007/s10567-026-00570-w (PMC13282340; doi:10.1007/s10567-026-00570-w)
Supplement: Supplementary file 1 — Supplementary Material [file 10567_2026_570_MOESM1_ESM.pdf]

Clinical Child and Family Psychology Review

A Scoping Review of Australian Aboriginal Early Relational Health Knowledge Systems

Shannon McNeair[1], Catherine Chamberlain[2], Naomi Priest[2,3,5], Tracy Evans-Whipp[1*], Suzanne Vassallo[1], Kayla Mansour[1], Lisa Ritland[4], Craig A. Olsson[1,5], Juli Coffin[6-8] & the *Australian Early Relational Health Network*

*Corresponding author:

Dr Tracy Evans-Whipp, Senior Research Fellow, School of Psychology, Deakin University

Email: t.evanswhipp@deakin.edu.au

Online Resource 1
Search Syntax for Database Search

Concept 1 – Outcome<br>Child & Family Relational Ecology<br>Reflects child exposure to relationships and observations of relationships with and between parents, siblings, grandparents, and caregivers	Concept 2 –<br>Indigenous terms
**[ti,ab]**<br>(Infan* OR child* OR fetal OR fetus OR foetal OR foetus OR "parent-child" OR "child-parent" OR "parent-infant" or "infant-parent" OR "fetus-parent" OR "parent-fetus" OR "foetus-parent" OR "parent-foetus" OR "mother-child" OR "child-mother" OR "mother-infant" or "infant-mother" OR "fetus- mother" OR "mother-fetus" OR "foetus-mother" OR "parent-child" OR "father-child" OR "child-father" OR "father-infant" or "infant-father" OR "fetus-father" OR "father-fetus" OR "foetus-father" OR "father-foetus" OR "maternal-child" OR "child-maternal" OR "maternal-infant" or "infant-maternal" OR "fetus-maternal" OR "maternal-fetus" OR "foetus-maternal" OR "maternal-foetus" OR "paternal-child" OR "child-paternal" OR "paternal-infant" or "infant-paternal" OR "fetus-paternal" OR "paternal-fetus" OR "foetus-paternal" OR "paternal-foetus" OR "parental-child" OR "child-parental" OR "parental-infant" or "infant-parental" OR "fetus-parental" OR "parental-fetus" OR "foetus-parental" OR "parental-foetus")<br>**N1 <u>(attach* OR relation* OR interact* OR bond*)</u>**<br><br>OR<br><br>(Grandparent* OR grandmother* OR grandfather* OR parent* OR maternal OR paternal OR father* OR mother* OR caregiv* OR alloparent* OR cousin* OR Aunt* OR Uncle* OR Nanny OR Nannies OR "early childcare" OR "early childhood educator" OR friend* OR peer* OR playmate* OR 'play mate*' OR 'class mate* OR classmate*) **N1** (**<u>sensitivity OR involve* OR relation* OR interact* OR</u>**	**[ti,ab]**<br>aboriginal* OR "first nation*" OR indigenous OR "torres strait" OR Murri OR Nyoongah OR Koori OR Goori OR Koorie OR Yolngu OR Anangu OR Palawa OR Nunga OR Ngarrindjeri OR "Murray Island" OR "Mer Island"

**investment* OR bond* OR attach* OR availab* OR responsiv* OR warm* OR hostil* OR connect*)**<br><br>OR<br><br>(Brother OR Sister OR Sibling) **N1 (Relations* OR conflict* OR interact*)**<br><br>OR<br><br>**“dyadic synchrony”**<br>**OR “dyadic attunement”**<br>**OR “dyadic mutuality”**<br>**OR Kinship**<br><br>OR<br><br>**‘Parent-parent’**<br>**OR Interparent***<br>**OR “parenting”**<br>**OR “parent* styles”**<br>**OR “parent* quality”**<br>**OR “parent* behav*”**<br>**OR “parent* conflict”**<br>**OR coparent***<br>**OR “co-parent*”**<br><br>OR<br><br>**“Family conflict” OR “Family cohesion” OR “Family relations*” OR “Family function*”**<br>**OR “Family interact*” OR “Family involve*”**<br><br>OR<br><br>**“Dyadic adjustment” OR “Relationship quality” OR “relationship conflict” OR “relationship satisfaction” OR “Marital quality” OR “marital conflict” OR “Marital satisfaction”**<br><br>OR<br><br>**“Social network analys*” OR**<br>**Egocentr* OR**<br>**“Whole network*” OR**<br>**“Network analys*” OR**<br>**“Ego network*” OR**<br>**Egonetwork* OR**<br>**Sociometr* OR**<br>**Sociogram* OR**<br>**Sociomap* OR**	

Sociocentr* OR<br>“Graph theory” OR<br>“Structural network*”	
